# Familial interstitial lung disease: emerging insights into screening and genetic risk

**DOI:** 10.3389/fmed.2026.1747200

**Published:** 2026-02-17

**Authors:** Ana Paola Hernández Cristancho, Lurdes Planas-Cerezales, Maria Molina-Molina, Raphael Borie, Wim A. Wuyts, Meritxell Jodar, Jacobo Sellares, Fernanda Hernandez-Gonzalez

**Affiliations:** 1 Department of Respiratory Medicine, Respiratory Institute, Hospital Clinic, Barcelona, Spain; 2 Department of Respiratory Medicine, Hospital de Viladecans, Viladecans, Spain; 3 ILD Unit, Department of Respiratory, Biomedical Research Institute of Bellvitge (IDIBELL), University Hospital of Bellvitge, Barcelona, Spain; 4 University of Barcelona, Barcelona, Spain; 5 Centro Investigación Biomédica en Red Enfermedades Respiratorias (CIBERES), Barcelona, Spain; 6 Université Paris Cité, UMR Inserm, CRI, Paris, France; 7 Service de Pneumologie Allergologie et Transplantation, Hôpital Bichat, AP-HP, Centre Constitutif du Centre de Référence des Maladies Pulmonaires Rares, FHU INFIRE, Paris, France; 8 Unit for Interstitial Lung Diseases, Department of Respiratory Medicine, University Hospitals Leuven, Leuven, Belgium; 9 Department of Biochemistry and Molecular Genetics, CDB, Hospital Clínic de Barcelona, Barcelona, Spain; 10 Instituto de Investigaciones Biomédicas August Pi i Sunyer (IDIBAPS), Barcelona, Spain

**Keywords:** familial pulmonary fibrosis, genetic screening, idiopathic pulmonary fibrosis, interstitial lung abnormalities, interstitial lung disease

## Abstract

Familial pulmonary fibrosis (FPF) is increasingly recognized as a distinct entity within the spectrum of interstitial lung diseases (ILDs), characterized by a significant genetic contribution involving genetic variation telomere-related genes, surfactant protein genes, and the *MUC5B* promoter polymorphism. These variants influence disease susceptibility, clinical course, and prognosis. Moreover, high-resolution computed tomography (HRCT) has revealed interstitial lung abnormalities (ILAs) as early manifestations in at-risk relatives, particularly amongst individuals with pathogenic variants, highlighting its central role in early detection. Despite substantial progress, significant challenges persist, particularly regarding the unidentified genetic variants in a considerable proportion of cases and the psychosocial impact associated with familial screening. Some studies suggest that HRCT-based surveillance from age 50 and genetic testing in affected individuals. Looking ahead, integrative approaches combining genetic, radiologic, functional, and biomarker data may enhance risk stratification and enable early intervention, moving towards a paradigm where FPF becomes a preventable condition rather than a relentlessly treatable progressive disease. This review addresses FPF, integrating advances in genetics, radiology, and clinical management. It highlights key developments in telomere biology, surfactant genes, and *MUC5B*, and discusses evidence-based strategies for screening and prevention, providing relevant insights for clinicians and researchers in ILD.

## 1 Introduction

Interstitial lung diseases (ILDs) encompass a heterogeneous group of disorders characterised by variable degrees of inflammation and fibrosis of the pulmonary interstitium (1). Despite their clinical, radiological, and physiological overlap (2), their aetiology is diverse. Environmental exposures and autoimmune conditions are well-recognised risk factors, but genetic predisposition has emerged as a key determinant of disease susceptibility and progression (3). Within this evolving landscape, interstitial lung abnormalities (ILAs) have been identified as an important radiological entity, defined as non-dependent, bilateral parenchymal abnormalities detected on high-resolution computed tomography (HRCT), including ground-glass or reticular opacities, lung distortion, traction bronchiectasis, and/or honeycombing involving more than 5% of a lung zone by visual assessment, but not meeting the diagnostic criteria for ILD (Table 1) (4, 5).

**Table 1:** Definition of interstitial lung disease (ILD) in those patients with interstitial lung abnormalities (ILA).

Domain	Criteria
Symptoms	Any amount of dyspnoea and/or cough that a clinician attributes to ILD
Physiology	Any abnormality in FVC, TLC, or DLCO attributed to ILD (value or *z*-score below the lower limit of normal)
	Meets physiologic criteria for progressive pulmonary fibrosis attributed to ILD
Imaging (HRCT scan)	Fibrotic abnormalities (honeycombing and/or reticulation with traction bronchiectasis) involving ≥5% of total lung volume by visual estimate
	Progressive fibrotic abnormality on serial HRCT
	Presence of a major fibrotic ILD pattern (UIP/probable UIP, fibrotic HP, fibrotic NSIP)
Pathology	Presence of a major fibrotic ILD pattern (UIP/probable UIP, fibrotic HP, fibrotic NSIP)

The diagnosis of ILD requires the presence of at least one of the following criteria. Adapted from Podolanczuk et al. (67).

Additional diagnostic evaluation may be necessary to define the specific ILD subtype, as the diagnosis of non-fibrotic ILD requires the integration of clinical, radiologic, and pathological domains.

HRCT, high-resolution computed tomography; HP, hypersensitivity pneumonitis; ILA, interstitial lung abnormality; ILD, interstitial lung disease; NSIP, nonspecific interstitial pneumonia; UIP, usual interstitial pneumonitis.

Idiopathic pulmonary fibrosis (IPF) was long regarded as a sporadic condition. Yet, over the past two decades, accumulating evidence of familial clustering has reshaped this paradigm. Approximately 20–25% of patients with IPF report a family history of ILD, while 5–10% of those with fibrotic non-IPF ILDs—such as hypersensitivity pneumonitis (HP) or connective tissue disease-associated ILD (CTD-ILD)—also have affected relatives (6). The recognition of familial pulmonary fibrosis (FPF) has been reinforced by landmark genetic discoveries, including the *MUC5B* promoter polymorphism, strongly associated with ILD susceptibility with usual interstitial pneumonia (UIP) pattern, and telomere-related gene (TRG) variants recognised as the most frequent monogenic cause (7, 8). These insights have deepened our understanding of pathogenesis and raised expectations for targeted therapeutic strategies (6).

Nevertheless, major uncertainties remain. Up to three-quarters of patients with FPF lack an identifiable variant, and no universally accepted genetic panel is standardized for clinical use. Furthermore, the predictive value of genetic findings for therapeutic response is largely unexplored, and strategies for screening at-risk relatives remain fragmented and controversial. Beyond the well-established associations, new susceptibility variants continue to be identified, underlining the complexity of gene–environment interactions and the urgent need for integrative models that combine radiological, functional, genetic, and biomarker data.

Moreover, radiological concepts have also evolved. ILAs are now recognized as early manifestations in at-risk relatives, with 15–30% of asymptomatic first-degree relatives of IPF patients demonstrating ILAs (4, 9, 10). Subpleural fibrotic ILAs, particularly in individuals with TRG variants or the minor T-allele of the *MUC5B* promoter polymorphism *rs35705950*, are associated with a higher risk of progression to overt ILD (11). By contrast, pulmonary function tests such as spirometry and DLCO, though valuable for longitudinal monitoring, lack sensitivity for detecting early disease, underscoring the central role of HRCT in screening strategies of patients with FPF (5).

In this review, we summarise current knowledge of the genetic basis of FPF, highlight emerging concepts in risk stratification, and critically discuss the implications for screening, early diagnosis, and clinical management of at-risk relatives, with an emphasis on current challenges and future opportunities.

## 2 Familial pulmonary fibrosis and ILA: a conceptual framework

FPF is broadly defined as the presence of fibrotic ILD in an individual together with at least one first- or second-degree relative affected by a fibrotic phenotype (12). Although HRCT is essential for ILD phenotyping, familial cases may display a broad spectrum of classic radiographic patterns (13, 14).

**Figure 1:**
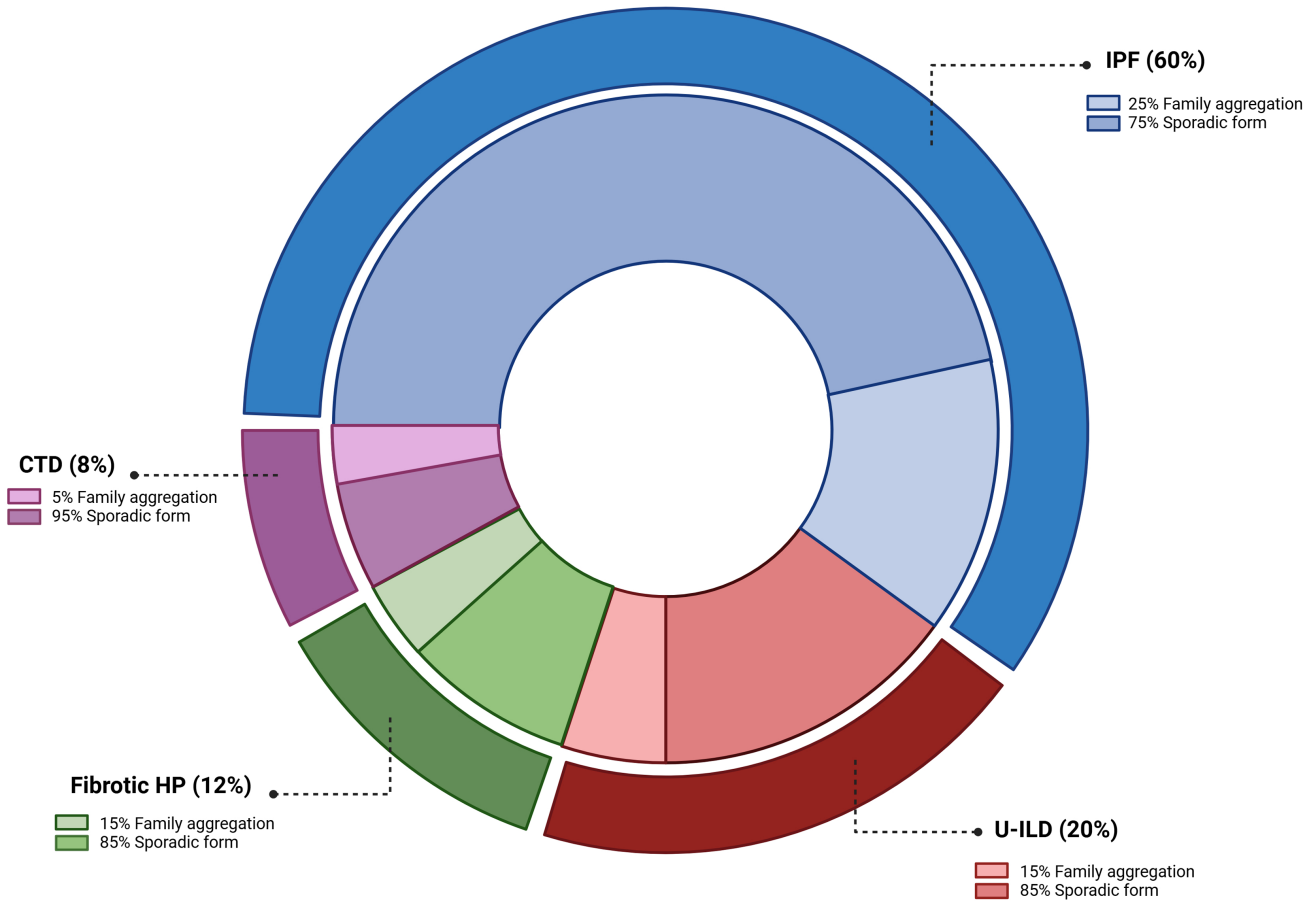
Clinical spectrum of diagnoses amongst patients with familial pulmonary fibrosis (FPF). FPF most frequently presents with an idiopathic pulmonary fibrosis (IPF) phenotype, though other ILD subtypes are also observed. HP, hypersensitivity pneumonitis; CTD, connective tissue disease; U-ILD, unclassifiable interstitial lung disease. Adapted from Zhang and Newton (73) created in https://BioRender.com.

IPF is the most frequent phenotype within FPF (14), but other forms—including fibrotic HP, CTD-ILD, and unclassifiable ILD (U-ILD)—are also represented (Figure 1). Familial ILDs are typically associated with an earlier age of onset and a more aggressive clinical course compared with sporadic cases (15). While a positive family history alone is insufficient to establish a diagnosis, it carries significant prognostic implications (6). In another paper, it is suggested that the disease trajectory is similar with a certain family, pointing even more towards FPF as a strong prognostic factor (16).

Beyond well-characterised familial clusters, first-degree relatives of patients with sporadic IPF also have an increased risk of ILAs. Although bilateral findings are required by definition, unilateral abnormalities may also be clinically relevant in selected high-risk populations. In particular, individuals with a strong family history of ILD or individuals with known pathogenic variants may be at increased risk of progression to ILD even when HRCT changes are unilateral (5, 17).

Three major ILA subcategories are recognised: (1) *nonsubpleural ILAs:* abnormalities without predominant subpleural localization; (2) *subpleural non-fibrotic ILAs:* abnormalities with predominant subpleural localisation but no evidence of fibrosis; and (3) *subpleural fibrotic ILAs:* abnormalities with predominant subpleural localization and imaging evidence of pulmonary fibrosis (18, 19). In both familial and sporadic IPF cohorts, 15–30% of asymptomatic first-degree relatives demonstrate ILAs on HRCT (4, 9, 10). This finding is strongly associated with older age, a history of smoking, the gain-of-function *MUC5B* T minor allele *rs35705950* and shortened peripheral blood leukocyte telomere length (4, 5, 10, 20).

## 3 Genetic basis of familial ILD

The strongest evidence for a genetic predisposition to ILD comes from familial clustering, particularly in homozygous twins raised apart (21), multigenerational families (22, 23), and genetically related relatives (12). In FPF, disease susceptibility arises from the combined influence of common and rare genetic variants, estimated to account for approximately 12.4% and 25–30% of overall risk, respectively (24). There is a wide range between rare genetic variants with large effect sizes, typically associated with monogenic (Mendelian) disorders, and frequent genetic variants with smaller effect sizes that underlie polygenic or complex diseases (25). Rare gene variants identified through candidate gene and family-based studies predominantly affect pathways involved in telomere maintenance, surfactant metabolism, and mitotic spindle assembly. Importantly, a single gene may harbour multiple disease-associated variants that can be categorized as either common or rare.

Pathogenic variants in TRG and surfactant protein genes represent the most consistent genetic determinants of FPF. In parallel, common variants, particularly in *MUC5B* (T minor allele *rs35705950*) and *TOLLIP* (including *rs111521887* and *rs3750920T*), have been shown to confer susceptibility across several ILD phenotypes with UIP pattern, including IPF and rheumatoid arthritis-associated ILD (RA-ILD) (26), and importantly, both variants are not only associated with IPF susceptibility but also with better survival in IPF patients (27).

**Figure 2:**
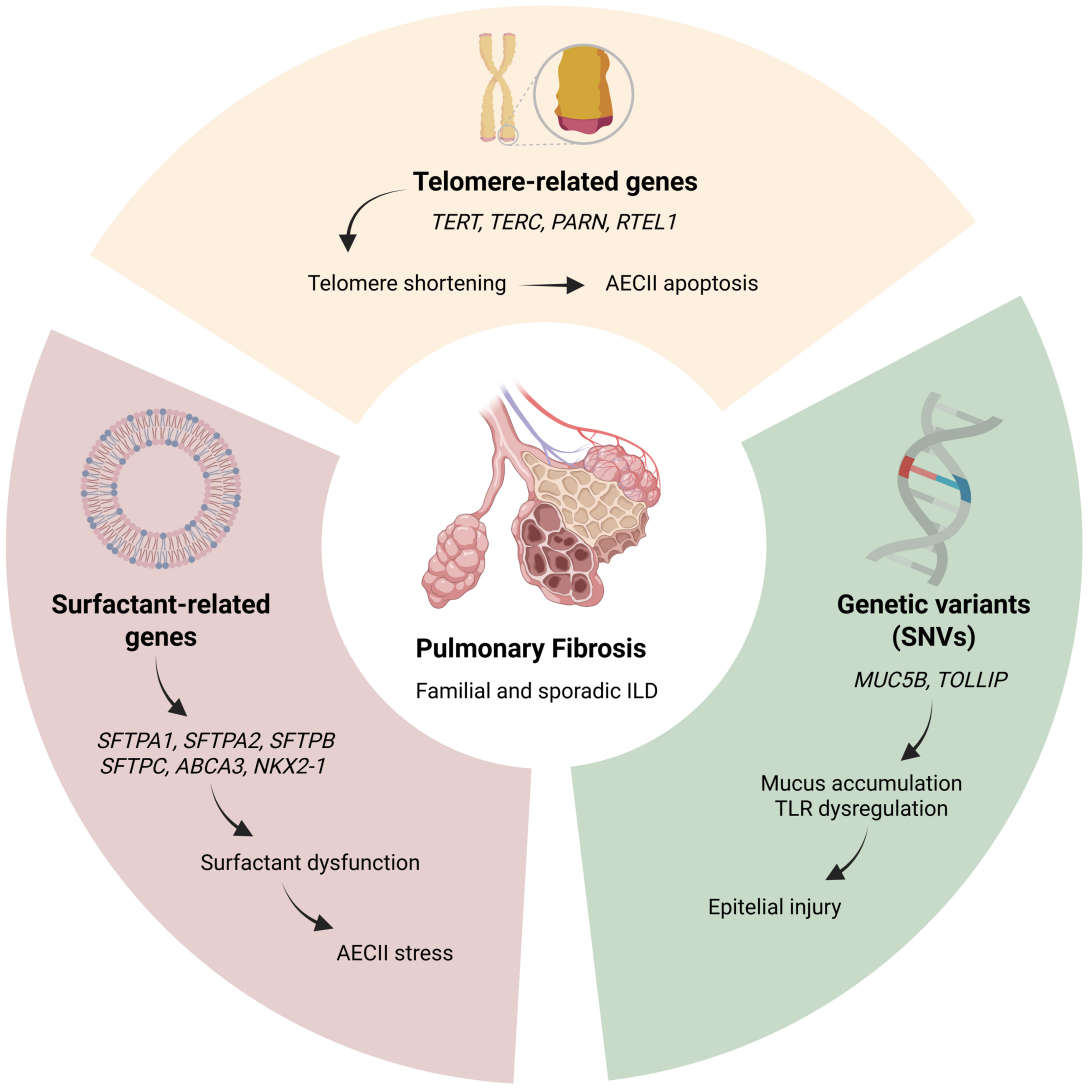
Genetic basis of familial ILD (74). AECII, alveolar type II cell; TLR, Toll-like receptor. Created in https://BioRender.com.

In addition, genome-wide association studies (GWAS) suggest that common single-nucleotide polymorphisms (SNPs) contribute roughly 5–15% of the genetic liability for IPF in the general population (28), implicating genes related to telomere biology, alveolar epithelial barrier integrity, and host defense mechanisms, as shown in Figure 2. Beyond inherited variation, epigenetic processes—including DNA methylation, histone modifications, and non-coding RNAs such as miR-21 and miR-29—modulate fibrotic pathways, thereby influencing disease initiation, progression, and phenotypic heterogeneity (29). Despite these advances, a substantial portion of the genetic risk underlying IIPs remains unexplained.

### 3.1 Telomere dysfunction

Telomeres are six-nucleotide repeats located at the ends of chromosomes that prevent DNA degradation and fusion. The enzyme telomerase preserves this structure by maintaining and elongating telomeres. Increasing evidence indicates that telomere shortening and the consequent genomic instability are central drivers of fibrotic processes (30, 31). Although telomere attrition occurs as part of normal ageing, pathogenic variants in TRG accelerate this process and often result in premature shortening. Furthermore, different environmental factors may induce telomere attrition such as smoking and radiation. Once telomere length falls below a critical threshold—defined as under the 10th percentile for age (32)—DNA damage responses are triggered, leading to apoptosis (33), particularly in alveolar epithelial type II cells (AECII) (34).

The clinical spectrum associated with TRG variants is heterogeneous. Approximately 50% of individuals with TRG variants develop IPF, while others present with fibrotic HP (7–12%), CTD-ILD (2–3%), U-ILD (8–20%), or other idiopathic interstitial pneumonias (14–18%) (35, 36). Overall, TRG variants are identified in roughly one-quarter of FPF kindreds, with *TERT* variants accounting for 8–15% of cases (8, 36, 37), *PARN* and *RTEL1* for 5–10% each (38), and *TERC* for 1–2% (36).

Importantly, short age-adjusted telomere length is not restricted to familial disease. It is found in around 50% of patients with FPF (38, 39), but also in sporadic ILD, including 20–60% of IPF (40, 41), 20–35% of fibrotic HP (42), and approximately 26% of RA-ILD (43). The mechanism leading to short telomere in those patients might include frequent variant in non-TRG and environmental risks factors (44). These findings underscore telomere dysfunction as a convergent pathogenic mechanism that extends across both familial and sporadic forms of ILD. Current evidence suggests that environmental exposures, particularly fine particulate air pollution (PM2.5), adversely affect patients with telomere dysfunction by promoting oxidative stress, genomic instability, and accelerated telomere shortening, thereby contributing to cellular senescence and pulmonary fibrosis progression. Recent studies further demonstrate associations between PM2.5 exposure, epigenetic alterations, and increased mortality in patients with fibrosing ILD (45).

Beyond the lung, telomere-related disorders exhibit incomplete penetrance and variable expressivity, which likely reflect the combined influence of environmental stressors, genetic background, and stochastic factors. A genetic anticipation phenomenon—characterized by earlier onset and more severe manifestations in successive generations—has also been observed in telomere biology disorder (TBD) due to progressive telomere shortening. Clinically, these entities are now recognized as part of a TBD, encompassing pulmonary fibrosis that may coexist with extrapulmonary involvement of variable severity, ranging from subtle laboratory abnormalities (such as premature greying, cytopenias or elevated liver enzymes) to overt organ failure (e.g., bone marrow failure, myelodysplastic syndrome/acute myeloid leukaemia or hepatic cirrhosis) (46).

### 3.2 Surfactant related genes

Beyond their essential role in maintaining alveolar stability, surfactant proteins contribute to pulmonary host defense by regulating immune responses and inflammation. They constitute up to 10% of total surfactant weight and include five key proteins: SP-A1, SP-A2, SP-B, SP-C, and SP-D, encoded by *SFTPA1*, *SFTPA2*, *SFTPB*, *SFTPC*, and *SFTPD*, respectively. Gene variants in *SFTPA1*, *SFTPA2*, *SFTPB*, and *SFTPC* are recognised monogenic causes of ILD and lung cancer (25, 47, 48).

Amongst these, *SFTPB* variants most frequently lead to severe neonatal respiratory failure, whereas *SFTPA1*, *SFTPA2*, and *SFTPC* genetic variation are implicated in both pediatric and adult ILD, particularly pulmonary fibrosis (49). In addition, gene variant involved in surfactant lipid transport, such as *ABCA3* (ATP binding cassette subfamily A member 3), and regulatory genes including *NKX2-1* (encoding thyroid transcription factor 1, TTF1), which governs transcription of both surfactant proteins and *ABCA3*, have also been identified (12). Collectively, these findings highlight disruption of surfactant homeostasis as a distinct group of monogenic causes of ILD (49).

From a clinical perspective, the identification of a rare surfactant gene variant in a proband has implications for family members. Genetic testing and radiological screening of unaffected individuals with pathogenic variants may enable early recognition of subclinical disease, providing opportunities for timely intervention and counselling (50).

### 3.3 MUC5B and disease susceptibility

Mucins (MUC) are gel-forming glycoproteins that constitute key components of airway mucus. The human genome encodes 17 mucins, of which MUC5AC and MUC5B are the most abundantly expressed in the airways (51). *MUC5B*, located on chromosome 11, encodes mucin 5 subtype B, a glycoprotein secreted by submucosal glands and airway secretory cells (51). It plays a central role in mucus production, mucociliary clearance, host defense, and the maintenance of airway and lung homeostasis (52).

Aberrant expression of MUC5B has been implicated in several respiratory diseases, including chronic obstructive pulmonary disease (COPD), asthma, cystic fibrosis (53), and severe community-acquired pneumonia (54). In the context of pulmonary fibrosis, excessive MUC5B production promotes mucus accumulation in the distal airways and impairs mucociliary clearance. This accumulation favours the retention of inhaled particles—such as cigarette smoke and air pollutants, both recognised risk factors for IPF—and contributes to epithelial injury. Moreover, excess MUC5B disrupts surfactant function and hampers AECII cell repair mechanisms (7). Collectively, these processes foster persistent epithelial damage, fibroproliferation, and honeycomb cyst formation, which are hallmark features of progressive pulmonary fibrosis (55).

Moreover, the *MUC5B* promoter variant *rs35705950* is a strong genetic risk factor for rheumatoid arthritis-associated ILD (RA-ILD), particularly in patients with a UIP pattern and in familial forms of ILD. While this variant does not increase susceptibility to rheumatoid arthritis itself, it confers a markedly increased risk of ILD amongst RA patients, with effect sizes comparable to those observed in IPF (56).

### 3.4 Toll-interacting protein (TOLLIP)

*TOLLIP* encodes a ubiquitin-binding protein that regulates multiple steps of Toll-like receptor (TLR) signalling. It is expressed across diverse lung cell populations—including monocytes, macrophages, regulatory T cells, and alveolar epithelial type I cells—in both healthy individuals and patients with IPF (57). Four isoforms (A–D) have been identified; three are expressed in human mononuclear cells, where they modulate innate immune responses and influence epithelial cell apoptosis (57).

Through its roles in inflammation regulation, autophagy, and vacuolar trafficking, *TOLLIP* has been implicated in a broad range of pulmonary diseases, including IPF, COPD, asthma, and respiratory infections (57). In the context of IPF, SNPs within the *TOLLIP* locus on chromosome 11 have been associated not only with disease susceptibility but also with prognosis (58). These findings suggest that *TOLLIP* functions as both a regulator of innate immunity and a genetic determinant of interindividual variability in fibrotic lung disease.

Genetic variation in *TOLLIP* has been implicated in both susceptibility to pulmonary fibrosis and disease progression. While the minor C allele of *TOLLIP rs5743890* appears protective against ILD development, it is paradoxically linked to worse survival and faster progression in established IPF. Other variants (*rs111521887G, rs3750920T*) increase ILD risk, though their prognostic impact is less consistent. Mechanistically, risk alleles are associated with reduced TOLLIP expression, enhanced epithelial apoptosis, and dysregulated innate immunity, promoting fibrotic remodelling (26).

Importantly, *TOLLIP* also has pharmacogenetic relevance, as IPF patients with the *rs3750920 TT* genotype benefit from N-acetylcysteine, whereas those with the CC genotype may experience harm, supporting a potential role for genotype-guided therapy; no such association has been observed with antifibrotic agents (59). In contrast, no association has been demonstrated between TOLLIP variants and response to antifibrotic agents such as nintedanib or pirfenidone (60).

Collectively, these findings indicate that *TOLLIP* genetic variants influence both disease susceptibility and progression in pulmonary fibrosis and may have therapeutic implications for NAC treatment, though not for antifibrotic therapies.

## 4 Clinical implications for at-risk relatives

One of the most pressing and unresolved challenges in FPF lies in defining appropriate clinical strategies for the relatives of affected individuals. Family members often express a strong interest in assessing their personal risk for developing ILD. However, despite increasing scientific attention, insufficient understanding prevents reliable predictions about individual susceptibility, timing of disease onset, or the mechanisms driving its pathogenesis—thereby limiting opportunities for proactive health management.

Although there is growing support for HRCT scan-based screening in asymptomatic relatives (12, 61), no consensus exists regarding fundamental aspects such as the optimal age to initiate screening, the frequency of follow-up, or the management of positive findings. Cohort studies suggest that 14–25% of asymptomatic relatives, at a mean age of approximately 50 years, already exhibit ILA (4, 5). Established risk factors—including older age, smoking, and shortened telomere length—contribute to risk stratification (9, 10, 62), but remain insufficient for precise prediction. Alarmingly, about 20% of these individuals progress to extensive ILA or clinical ILD within 5 years, despite having preserved pulmonary function at baseline (5). This underscores the limited sensitivity and specificity of pulmonary function testing as a screening tool, even though such tests remain valuable for longitudinal monitoring.

Genetic insights further highlight the complexity of FPF. Despite advances in sequencing technologies, up to 75% of patients lack an identifiable pathogenic variant. Moreover, even when gene variants are detected, the genetically complex and multifactorial nature of the disease makes the dichotomy of ‘with’ versus ‘without pathogenic variants’ misleading. Consequently, most families occupy an intermediate zone in which genetic information provides limited predictive value.

Together, these observations reveal a critical gap: current approaches are fragmented, and neither radiology nor genomics in isolation can provide the clarity that families seek. What is urgently needed is an integrated, evidence-based framework that bridges imaging, genetics, and clinical monitoring to truly inform decision-making in at-risk populations.

### 4.1 Screening challenges and preventive approaches in FPF

An important consideration in screening programmes for FPF is the potential impact on asymptomatic individuals. The identification of radiological, genetic, or biological abnormalities has been associated with feelings of regret and increased psychological burden amongst relatives who undergo screening (63). Consequently, any screening initiative should ideally be supported by appropriate multidisciplinary approach, including emotional, and decisions regarding testing must be accompanied by transparent and understandable about the implications of potential outcomes.

While prospective studies in FPF relatives are still lacking, practical recommendations can nonetheless be offered. These include minimising modifiable risk factors such as smoking, occupational exposures, and air pollution, as well as ensuring that vaccinations against respiratory pathogens are up to date. Indeed a recent prospective study showed that most relatives have at least one harmful respiratory exposure that may modified after genetic counselling (64). Although such measures do not replace future disease-modifying therapies, they may help reduce the risk of disease development or progression and remain relevant for improving overall health and resilience.

Genetic testing is most strongly indicated in patients with familial ILD, particularly those with two or more biologically related affected relatives, early-onset disease (<50 years), unusually severe or rapidly progressive disease, or syndromic features suggestive of a TBD. When testing is undertaken, the appropriate strategy is to begin with the index case or, in its absence, with an affected family member, as this increases the likelihood of identifying a segregating pathogenic variant. Testing unaffected relatives in the absence of this step may yield results that are challenging to interpret and of limited clinical value. It is important to recognise that only a positive genetic test result is unequivocally informative, but a negative result does not exclude genetic risk. To maximise diagnostic yield, testing should be reserved for patients with a high pre-test probability, as defined by clinical features and family history (65).

Importantly, screening strategies in FPF should not be limited to the detection of fibrosing lung disease. Given the multisystemic nature of telomere-related disorders, evaluation should also include potential extrapulmonary manifestations that may precede or accompany pulmonary fibrosis. Recognising these systemic features can improve early identification of at-risk individuals and refine risk stratification within affected families.

Moreover, about one-third of lung transplant recipients with PPF have telomere-dysfunction related to pathogenic TRG variants and/or short telomere length, and although carefully selected patients do not show consistently worse short- or mid-term outcomes compared with non-TRG variants/telomere shortening recipients, emerging evidence suggests that post-transplant care and treatment management may be more complex—given higher rates of complications such as CMV infection, cytopenias, and anastomotic problems—though definitive conclusions remain limited by heterogeneous data (66).

A positive result carries several potential implications. It may influence therapeutic decisions, such as transplant timing or the initiation of antifibrotic therapy; guide targeted screening of at-risk relatives; and support referral for specialised genetic counselling. These benefits must, however, be balanced against the limited variant interpretation, and the psychological impact of results. Together, these considerations highlight the need for multidisciplinary decision-making and careful integration of genetic testing into the broader clinical context of ILD.

In essence, successful FPF screening requires an integrated approach that combines clinical vigilance, preventive strategies, genetic counselling and psychological support, ensuring that early detection truly translates into meaningful patient benefit.

### 4.2 Evidence-based suggestions for screening

Given the growing recognition of FPF and the increasing availability of advanced imaging and genetic tools, the question of how and when to screen at-risk relatives has gained clinical relevance. Although high-quality evidence remains limited, recent studies and expert consensus provide a rationale for pragmatic, evidence-informed screening approaches.

A meticulous collection of the family history—ideally covering at least three generations—is a cornerstone of FPF evaluation. A detailed pedigree allows clinicians to identify inheritance patterns, anticipate variable disease expression, and guide genetic testing strategies. Importantly, family histories should be updated at each clinical visit, as new cases of interstitial lung disease or related extrapulmonary manifestations may emerge over time, refining the assessment of familial risk.

In this regard, the American Thoracic Society clinical statement presented evidence-based suggestions for evaluation and management of adults with ILA and a first-degree relative with pulmonary fibrosis (67). The evidence-based suggestions shown in Table 2 aim to guide clinicians in identifying ILA or ILD at early, potentially reversible stages (4, 10, 14, 62, 68–70). Together, these recommendations provide support to clinicians by standardizing the approach to ILAs. Although there are several important unanswered questions, it gives a direction for future initiatives, while emphasizing the need to identify treatment options for ILA.

**Table 2:** Evidence-based recommendations for screening in FPF.

Domain, Criteria	Recommendations
Chest HRCT scan in relatives of FPF patients	For adults over 50 years with a **first-degree relative diagnosed with FPF**, chest HRCT scan screening is **suggested** to detect ILA or ILD, which are identified in approximately 26% of such individuals (7, 9, 13, 48, 51–53).
Chest HRCT scan in relatives of IPF patients	For adults over 50 years with a **first-degree relative affected by IPF**, but without other family members with ILD, current evidence remains limited.
	**No firm recommendation** for or against radiological screening can be made, although prevalence estimates for ILA are similar (around 24%) (7, 13, 52, 54).
Timing of screening	Screening is generally recommended to begin **around age 50, or earlier** if disease onset occurred at a younger age in family members.
	When the initial HRCT scan is **negative**, repeat screening at intervals of **no less than five years** is suggested (55).
MUC5B promoter variant testing	It has been strongly advised **against** the use of *MUC5B* promoter variant testing as an **initial screening** tool prior to chest HRCT scan in adults over 50 with a first-degree relative with FPF or IPF. The sensitivity (56%) and specificity (60%) of MUC5B are insufficient to replace or precede HRCT scan.
	Genetic testing for *MUC5B* may provide additional risk stratification **once ILA or ILD are identified** on HRCT scan images (55).
Telomere length measurement as initial screening	Telomere length assessment is **not recommended** as a first-line screening tool before chest HRCT in adults over 50 with a first-degree relative with pulmonary fibrosis. The sensitivity (50%) and specificity (72%) are inadequate for screening, and current evidence remains limited.
	Telomere length testing may be valuable **after HRCT-detected ILAs/ILD**, particularly for identifying individuals with multi-organ involvement (e.g., liver disease, bone marrow failure) and to guide targeted genetic testing (55).
Baseline telomere length in ILAs	Routine baseline telomere length testing in all patients with ILAs is **not recommended**, given inconsistent evidence for its prognostic value regarding progression or mortality, alongside concerns about cost, availability, and clinical utility.
	Testing may be appropriate in cases with a **family history** or clinical features suggestive of **telomeropathy** [e.g., premature hair greying, liver disease, unexplained cytopenia (55)].

HRCT, high-resolution computed tomography; FPF, familial pulmonary fibrosis; ILA, interstitial lung abnormalities; ILD, interstitial lung disease; IPF, idiopathic pulmonary fibrosis. The bold values is for highlight some important concepts.

### 4.3 Unresolved clinical challenges in asymptomatic relatives

The management of asymptomatic relatives of patients with FPF remains a major unresolved challenge. While many seek clarity regarding their individual risk, evidence is insufficient to define standardised protocols for screening or follow-up. Cohort studies indicate that a significant proportion of relatives may harbour ILA before symptoms emerge, yet predictors of progression are poorly understood (4, 14, 71). This uncertainty complicates decisions on the timing and intensity of surveillance and raises concerns about the psychological burden of monitoring individuals who may never develop clinically relevant disease.

### 4.4 Role of HRCT scan and lung function testing in early detection

HRCT scan is currently the most sensitive tool for detecting early ILD or ILA in at-risk relatives, though its optimal use remains debated. Critical questions—such as the appropriate age to begin screening, the interval between scans, and the management of indeterminate findings—remain unanswered. Moreover, HRCT entails radiation exposure and a risk of overdiagnosis or anxiety in otherwise healthy individuals (14). Emerging imaging technologies, including lung ultrasound and photon-counting computed tomography, may help address some of these limitations in the future. Pulmonary function tests, although insufficiently sensitive for early abnormalities detection, remain valuable for longitudinal monitoring once ILD is established (67).

### 4.5 Towards multimodal risk prediction and personalised approach

A central limitation of the current landscape is that no single modality—imaging, lung function testing, genetic analysis, or biomarker measurement—can independently provide accurate prediction of disease risk. Future advances in FPF management will likely rely on integrated, personalized models that combine radiologic, physiologic, genetic, and molecular data. Such multidimensional approaches may enable early identification of high-risk individuals, refine surveillance strategies, and pave the way for preventive or early therapeutic interventions in FPF.

Ongoing research is increasingly focused on the discovery of biomarkers to refine risk stratification and disease monitoring. Shortened telomere length has been associated with a higher prevalence of ILAs and systemic manifestations (e.g., liver disease, bone marrow failure), although its limited sensitivity, specificity, and technical complexity currently preclude routine clinical use. Circulating biomarkers such as KL-6 have shown promise as indicators of alveolar injury and fibrotic activity, but their predictive value in asymptomatic relatives remains to be validated.

Notably, the PRECISIONS trial, which randomized patients with IPF carrying the *TOLLIP rs3750920* TT genotype to receive oral *N*-acetylcysteine or placebo, demonstrated a pharmacogenetic interaction between *N*-acetylcysteine and the TOLLIP polymorphism, highlighting the critical need for molecularly guided, personalized therapeutic strategies and randomized clinical trials and representing an important step towards precision medicine in fibrotic ILD (72).

## 5 Conclusion

Genetic and epigenetic discoveries have deepened our understanding of ILD susceptibility, onset, and prognosis. However, no standardised genetic panel currently exists, and the therapeutic implications of these findings remain uncertain, highlighting the urgent need for further research and precision medicine approaches.

The screening of asymptomatic relatives continues to raise psychological and ethical concerns, underscoring the importance of counselling and transparent communication. Although antifibrotic agents are not yet suitable for preventive use, risk reduction strategies—such as smoking cessation, minimising environmental exposures, and maintaining appropriate vaccination—remain fundamental.

Looking ahead, the development of preventive therapies may transform ILD from a relentlessly progressive condition into one where early intervention and even prevention become possible, ultimately improving long-term outcomes for patients and their families.

## References

[1] Maher TM. Interstitial lung disease: a review. JAMA. (2024) 331:1655–65. doi: 10.1001/jama.2024.3669 38648021

[2] Travis WD Costabel U Hansell DM King TE Lynch DA Nicholson AG . An official American Thoracic Society/European Respiratory Society statement: update of the international multidisciplinary classification of the idiopathic interstitial pneumonias. Am J Respir Crit Care Med. (2013) 188:733–48. doi: 10.1164/rccm.201308-1483ST 24032382 PMC5803655

[3] Lederer DJ Martinez FJ. Idiopathic pulmonary fibrosis. N Engl J Med. (2018) 378:1811–23. doi: 10.1056/NEJMra1705751 29742380

[4] Hunninghake GM Quesada-Arias LD Carmichael NE Martinez Manzano JM de Frías SP Baumgartner MA . Interstitial lung disease in relatives of patients with pulmonary fibrosis. Am J Respir Crit Care Med. (2020) 201:1240–8. doi: 10.1164/rccm.201908-1571OC 32011908 PMC7233344

[5] Salisbury ML Hewlett JC Ding G Markin CR Douglas K Mason W . Development and progression of radiologic abnormalities in individuals at risk for familial interstitial lung disease. Am J Respir Crit Care Med. (2020) 201:1230–9. doi: 10.1164/rccm.201909-1834OC 32011901 PMC7233345

[6] Cutting CC Bowman WS Dao N Pugashetti JV Garcia CK Oldham JM . Family history of pulmonary fibrosis predicts worse survival in patients with interstitial lung disease. Chest. (2021) 159:1913–21. doi: 10.1016/j.chest.2021.01.026 33484728 PMC8173755

[7] Seibold MA Wise AL Speer MC Steele MP Brown KK Loyd JE . A common MUC5B promoter polymorphism and pulmonary fibrosis. N Engl J Med. (2011) 364:1503–12. doi: 10.1056/NEJMoa1013660 21506741 PMC3379886

[8] Armanios MY Chen JJL Cogan JD Alder JK Ingersoll RG Markin C . Telomerase mutations in families with idiopathic pulmonary fibrosis. N Engl J Med. (2007) 356:1317–26. doi: 10.1056/NEJMoa066157 17392301

[9] Kropski JA Pritchett JM Zoz DF Crossno PF Markin C Garnett ET . Extensive phenotyping of individuals at risk for familial interstitial pneumonia reveals clues to the pathogenesis of interstitial lung disease. Am J Respir Crit Care Med. (2015) 191:417–26. doi: 10.1164/rccm.201406-1162OC 25389906 PMC4351594

[10] Mathai SK Humphries S Kropski JA Blackwell TS Powers J Walts AD . MUC5B variant is associated with visually and quantitatively detected preclinical pulmonary fibrosis. Thorax. (2019) 74:1131–9. doi: 10.1136/thoraxjnl-2018-212430 31558622 PMC7535073

[11] Moll M Peljto AL Kim JS Xu H Debban CL Chen X . A polygenic risk score for idiopathic pulmonary fibrosis and interstitial lung abnormalities. Am J Respir Crit Care Med. (2023) 208:791–801. doi: 10.1164/rccm.202212-2257OC 37523715 PMC10563194

[12] Borie R Kannengiesser C Antoniou K Bonella F Crestani B Fabre A . European Respiratory Society statement on familial pulmonary fibrosis. Eur Respir J. (2023) 61:2201383. doi: 10.1183/13993003.01383-2022 36549714

[13] Duminy-Luppi D Alcaide-Aldeano A Planas-Cerezales L Bermudo G Vicens-Zygmunt V Luburich P . Diagnostic and prognostic implications of family history of fibrotic interstitial lung diseases. Respir Res. (2024) 25:433. doi: 10.1186/s12931-024-03063-y 39695595 PMC11656921

[14] McGroder CF Zhang D Choudhury M Podolanczuk AJ Lederer D Hoffman EA . Radiographic lung abnormalities in first-degree relatives of patients with different subtypes of pulmonary fibrosis. Chest. (2023) 163:1471–5. doi: 10.1016/j.chest.2023.01.012 36649755 PMC10258432

[15] Peabody JW Hayes EW. Idiopathic pulmonary fibrosis: its occurrence in identical twin sisters. Dis Chest. (1950) 18:330–44. doi: 10.1016/s0096-0217(15)34710-5 14778377

[16] Goos T Dubbeldam A Vermant M Gogaert S De Sadeleer LJ De Crem N . Intrafamilial correlation and variability in the clinical evolution of pulmonary fibrosis. Chest. (2023) 164:1476–80. doi: 10.1016/j.chest.2023.07.003 37437878

[17] Salisbury ML Markin C Fadely T Guttentag AR Humphries SM Lynch DA . Progressive early interstitial lung abnormalities in persons at risk for familial pulmonary fibrosis: a prospective cohort study. Am J Respir Crit Care Med. (2024) 210:1441–52. doi: 10.1164/rccm.202403-0524OC 39137317 PMC11716039

[18] Hata A Hino T Li Y Johkoh T Christiani DC Lynch DA . Traction bronchiectasis/bronchiolectasis in interstitial lung abnormality: follow-up in the COPDGene study. Am J Respir Crit Care Med. (2023) 207:1395–8. doi: 10.1164/rccm.202211-2061LE 36898128 PMC10595461

[19] Hida T Nishino M Hino T Lu J Putman RK Gudmundsson EF . Traction bronchiectasis/bronchiolectasis is associated with interstitial lung abnormality mortality. Eur J Radiol. (2020) 129:109073. doi: 10.1016/j.ejrad.2020.109073 32480316 PMC7930316

[20] Steele MP Peljto AL Mathai SK Humphries S Bang TJ Oh A . Incidence and progression of fibrotic lung disease in an at-risk cohort. Am J Respir Crit Care Med. (2023) 207:587–93. doi: 10.1164/rccm.202206-1075OC 36094461 PMC10870916

[21] Spagnolo P Grunewald J du Bois RM. Genetic determinants of pulmonary fibrosis: evolving concepts. Lancet Respir Med. (2014) 2:416–28. doi: 10.1016/S2213-2600(14)70047-5 24815806

[22] Juge P-A Lee JS Ebstein E Furukawa H Dobrinskikh E Gazal S . MUC5B promoter variant and rheumatoid arthritis with interstitial lung disease. N Engl J Med. (2018) 379:2209–19. doi: 10.1056/NEJMoa1801562 30345907 PMC6371965

[23] Kropski JA. Familial interstitial lung disease. Semin Respir Crit Care Med. (2020) 41:229–37. doi: 10.1055/s-0040-1708054 32279293 PMC7272214

[24] Peljto AL Blumhagen RZ Walts AD Cardwell J Powers J Corte TJ . Idiopathic pulmonary fibrosis is associated with common genetic variants and limited rare variants. Am J Respir Crit Care Med. (2023) 207:1194–202. doi: 10.1164/rccm.202207-1331OC 36602845 PMC10161752

[25] Ng N Molina-Molina M Adegunsoye A Borie R Newton CA Raby B . Genetics of interstitial lung diseases: a state-of-the-art review. Eur Respir J. (2025) 66:2301079. doi: 10.1183/13993003.00788-2025 40841141

[26] Li X Cui B Jiang L. Associations between genetic variants of toll-interacting proteins and interstitial lung diseases: a systematic review and meta-analysis. Orphanet J Rare Dis. (2024) 19:418. doi: 10.1186/s13023-024-03410-8 39578840 PMC11583435

[27] Adegunsoye A Kropski JA Behr J Blackwell TS Corte TJ Cottin V . Genetics and genomics of pulmonary fibrosis: charting the molecular landscape and shaping precision medicine. Am J Respir Crit Care Med. (2024) 210:401–23. doi: 10.1164/rccm.202402-0346PP 38573068 PMC11351799

[28] Leavy OC Ma SF Molyneaux PL Maher TM Oldham JM Flores C . Proportion of idiopathic pulmonary fibrosis risk explained by known common genetic loci in European populations. Am J Respir Crit Care Med. (2021) 203:775–8. doi: 10.1164/rccm.202008-3211LE 33226834 PMC7958523

[29] Valand A Rajasekar P Wain LV Clifford RL. Interplay between genetics and epigenetics in lung fibrosis. Int J Biochem Cell Biol. (2025) 180:106418. doi: 10.1016/j.biocel.2025.106739 39848439

[30] Martinez FJ Collard HR Pardo A Raghu G Richeldi L Selman M . Idiopathic pulmonary fibrosis. Nat Rev Dis Primers. (2017) 3:17074. doi: 10.1038/nrdp.2017.74 29052582

[31] Selman M López-Otín C Pardo A. Age-driven developmental drift in the pathogenesis of idiopathic pulmonary fibrosis. Eur Respir J. (2016) 48:538–52. doi: 10.1183/13993003.00032-2016 27390284

[32] Spagnolo P Lee JS. Recent advances in the genetics of idiopathic pulmonary fibrosis. Curr Opin Pulm Med. (2023) 29:399–405. doi: 10.1097/MCP.0000000000000996 37410458 PMC10470435

[33] van Batenburg AA Kazemier KM van Oosterhout MFM van der Vis JJ Grutters JC Goldschmeding R . Telomere shortening and DNA damage in culprit cells of different types of progressive fibrosing interstitial lung disease. ERJ Open Res. (2021) 7:00128–2021. doi: 10.1183/23120541.00691-2020 PMC8165375 34084786

[34] Zhang K Xu L Cong YS. Telomere dysfunction in idiopathic pulmonary fibrosis. Front Med (Lausanne). (2021) 8:725950. doi: 10.3389/fmed.2021.739810 PMC8631932 34859008

[35] Newton CA Batra K Torrealba J Kozlitina J Glazer CS Aravena C . Telomere-related lung fibrosis is diagnostically heterogeneous but uniformly progressive. Eur Respir J. (2016) 48:1710–20. doi: 10.1183/13993003.00308-2016 27540018 PMC5433348

[36] Borie R Tabèze L Thabut G Nunes H Cottin V Marchand-Adam S . Prevalence and characteristics of TERT and TERC mutations in suspected genetic pulmonary fibrosis. Eur Respir J. (2016) 48:1721–31. doi: 10.1183/13993003.01037-2016 27836952

[37] Tsakiri KD Cronkhite JT Kuan PJ Xing C Raghu G Weissler JC . Adult-onset pulmonary fibrosis caused by mutations in telomerase. Proc Natl Acad Sci USA. (2007) 104:7552–7. doi: 10.1073/pnas.0701009104 17460043 PMC1855917

[38] Stuart BD Choi J Zaidi S Xing C Holohan B Chen R . Exome sequencing links mutations in PARN and RTEL1 with familial pulmonary fibrosis and telomere shortening. Nat Genet. (2015) 47:512–7. doi: 10.1038/ng.3278 25848748 PMC4414891

[39] Cronkhite JT Xing C Raghu G Chin KM Torres F Rosenblatt RL . Telomere shortening in familial and sporadic pulmonary fibrosis. Am J Respir Crit Care Med. (2008) 178:729–37. doi: 10.1164/rccm.200804-550OC 18635888 PMC2556455

[40] Zhang D Newton CA Wang B Povysil G Noth I Martinez FJ . Utility of whole genome sequencing in assessing risk and clinically relevant outcomes for pulmonary fibrosis. Eur Respir J. (2022) 60:2200801. doi: 10.1183/13993003.00577-2022 36028256 PMC10038316

[41] Alder JK Chen JJL Lancaster L Danoff S Su SC Cogan JD . Short telomeres are a risk factor for idiopathic pulmonary fibrosis. Proc Natl Acad Sci USA. (2008) 105:13051–6. doi: 10.1073/pnas.0804280105 18753630 PMC2529100

[42] Ley B Newton CA Arnould I Elicker BM Henry TS Vittinghoff E . The MUC5B promoter polymorphism and telomere length in patients with chronic hypersensitivity pneumonitis: an observational cohort-control study. Lancet Respir Med. (2017) 5:639–47. doi: 10.1016/S2213-2600(17)30216-9 28648751 PMC5555581

[43] Newton CA Oldham JM Ley B Anand V Adegunsoye A Liu G . Telomere length and genetic variant associations with interstitial lung disease progression and survival. Eur Respir J. (2019) 53:1801641. doi: 10.1183/13993003.01641-2018 30635297 PMC6612265

[44] Duckworth A Jackson L Green H Khan A Wang C Condescu A . Polygenic risk and rare variants in endotypes of idiopathic pulmonary fibrosis. medRxiv. (2025):25328177. doi: 10.1101/2025.05.22.25328177 PMC12952213 41616791

[45] Stanel SC Callum J Rivera-Ortega P. Genetic and environmental factors in interstitial lung diseases: current and future perspectives on early diagnosis of high-risk cohorts. Front Med (Lausanne). (2023) 10:1222203. doi: 10.3389/fmed.2023.1232655 PMC10435297 37601795

[46] Savage SA Bertuch AA Agarwal S Aubert G Beier F Bonfim C . Different phenotypes with different endings—telomere biology disorders and cancer predisposition with long telomeres. Br J Haematol. (2025) 206:69–73. doi: 10.1111/bjh.19141 39462986 PMC11739769

[47] Brudon A Legendre M Mageau A Bermudez J Bonniaud P Bouvry D . High risk of lung cancer in surfactant-related gene variant carriers. Eur Respir J. (2024) 63:2400284. doi: 10.1183/13993003.01809-2023 PMC11063619 38575158

[48] Raghu G Torres JM Bennett RL. Genetic factors for ILD—the path of precision medicine. Lancet Respir Med. (2024) 12:350–2. doi: 10.1016/S2213-2600(24)00114-5 38521082

[49] van Moorsel CHM van der Vis JJ Grutters JC. Genetic disorders of the surfactant system: focus on adult disease. Eur Respir Rev. (2021) 30:200266. doi: 10.1183/16000617.0085-2020 PMC9489129 33597124

[50] Crossno PF Polosukhin VV Blackwell TS Johnson JE Markin C Moore PE . Identification of early interstitial lung disease in an individual with genetic variations in ABCA3 and SFTPC. Chest. (2010) 137:969–73. doi: 10.1378/chest.09-1688 20371530 PMC2851554

[51] Fahy JV Dickey BF. Airway mucus function and dysfunction. N Engl J Med. (2010) 363:2233–47. doi: 10.1056/NEJMra0910061 21121836 PMC4048736

[52] Roy MG Livraghi-Butrico A Fletcher AA McElwee MM Evans SE Boerner RM . Muc5b is required for airway defence. Nature. (2014) 505:412–6. doi: 10.1038/nature12807 24317696 PMC4001806

[53] Williams OW Sharafkhaneh A Kim V Dickey BF Evans CM. Airway mucus: from production to secretion. Am J Respir Cell Mol Biol. (2006) 34:527–36. doi: 10.1165/rcmb.2005-0436SF 16415249 PMC2644218

[54] Fan L Lu Y Wang Y Zhang X Wu Y Sun H . Respiratory MUC5B disproportion is involved in severe community-acquired pneumonia. BMC Pulm Med. (2022) 22:89. doi: 10.1186/s12890-022-01870-x 35292003 PMC8922065

[55] Biondini D Cocconcelli E Bernardinello N Lorenzoni G Rigobello C Lococo S . Prognostic role of MUC5B rs35705950 genotype in patients with idiopathic pulmonary fibrosis on antifibrotic treatment. Respir Res. (2021) 22:98. doi: 10.1186/s12931-021-01694-z 33794872 PMC8017848

[56] Klein J Wheeler AM Baker JF Yang Y Roul P Frideres H . MUC5B promoter variant and survival in rheumatoid arthritis–associated interstitial lung disease. Rheumatology. (2025) 64:i36–43. doi: 10.1093/rheumatology/keae615 PMC12695044 39504460

[57] Li X Goobie GC Gregory AD Kass DJ Zhang Y. Toll-interacting protein in pulmonary diseases: abiding by the goldilocks principle. Am J Respir Cell Mol Biol. (2021) 64:536–46. doi: 10.1165/rcmb.2020-0470TR 33233920 PMC8086045

[58] Noth I Zhang Y Ma SF Flores C Barber M Huang Y . Genetic variants associated with idiopathic pulmonary fibrosis susceptibility and mortality: a genome-wide association study. Lancet Respir Med. (2013) 1:309–17. doi: 10.1016/S2213-2600(13)70045-6 24429156 PMC3894577

[59] Oldham JM Ma SF Martinez FJ Anstrom KJ Raghu G Schwartz DA . TOLLIP, MUC5B, and the response to N-acetylcysteine among individuals with idiopathic pulmonary fibrosis. Am J Respir Crit Care Med. (2015) 192:1475–82. doi: 10.1164/rccm.201505-1010OC 26331942 PMC4731723

[60] Isshiki T Koyama K Homma S Sakamoto S Yamasaki A Shimizu H . Association of rs3750920 polymorphism in TOLLIP with clinical characteristics of fibrosing interstitial lung diseases in Japanese patients. Sci Rep. (2021) 11:16202. doi: 10.1038/s41598-021-95869-9 34376770 PMC8355271

[61] Newton CA Oldham JM Applegate C Carmichael N Powell K Dilling D . The role of genetic testing in pulmonary fibrosis: a perspective from the Pulmonary Fibrosis Foundation genetic testing work group. Chest. (2022) 162:394–405. doi: 10.1016/j.chest.2022.03.030 35337808 PMC9424324

[62] Steele MP Speer MC Loyd JE Brown KK Herron A Slifer SH . Clinical and pathologic features of familial interstitial pneumonia. Am J Respir Crit Care Med. (2005) 172:1146–52. doi: 10.1164/rccm.200408-1104OC 16109978 PMC2718398

[63] Carmichael N Martinez Manzano JM Quesada-Arias LD Poli SDF Baumgartner MA Planchart Ferretto MA . Psychological impact of genetic and clinical screening for pulmonary fibrosis on asymptomatic first-degree relatives of affected individuals. Thorax. (2021) 76:621–3. doi: 10.1136/thoraxjnl-2020-216405 33483364 PMC8238310

[64] Sesé L Bouvry D Kannengiesser C Wémeau-Stervinou L Jouneau S Prevot G . Behavioral changes in exposure patterns after genetic counseling among asymptomatic first-degree relatives of patients with pulmonary fibrosis carrying a telomere-related gene variant. ERJ Open Res. (2023) 9:00341–2023. doi: 10.1183/23120541.01396-202536949965

[65] Kropski JA Young LR Cogan JD Mitchell DB Lancaster LH Worrell JA . Genetic evaluation and testing of patients and families with idiopathic pulmonary fibrosis. Am J Respir Crit Care Med. (2017) 195:1423–8. doi: 10.1164/rccm.201609-1820PP 27786550 PMC5470751

[66] Bordas-Martinez J Miedema JR Mathot BJ Seghers L Galjaard RJH Raaijmakers MHGP . Outcomes of lung transplantation in patients with telomere-related forms of progressive fibrosing interstitial lung disease: a systematic review. JHLT Open. (2024) 3:100083. doi: 10.1016/j.jhlto.2024.100054 PMC11935452 40145120

[67] Podolanczuk AJ Hunninghake GM Wilson KC Khor YH Kheir F Pang B . Approach to the evaluation and management of interstitial lung abnormalities: an official American Thoracic Society clinical statement. Am J Respir Crit Care Med. (2025) 211:1132–55. doi: 10.1164/rccm.202505-1054ST 40387336 PMC12264694

[68] Rosas IO Ren P Avila NA Chow CK Franks TJ Travis WD . Early interstitial lung disease in familial pulmonary fibrosis. Am J Respir Crit Care Med. (2007) 176:698–705. doi: 10.1164/rccm.200702-254OC 17641157 PMC1994234

[69] Rose JA Planchart Ferretto MA Maeda AH Perez Garcia MF Carmichael NE Gulati S . Progressive interstitial lung disease in relatives of patients with pulmonary fibrosis. Am J Respir Crit Care Med. (2023) 207:211–4. doi: 10.1164/rccm.202208-1470LE 36099425 PMC9893330

[70] Lucas SEM Raspin K Mackintosh J Glaspole I Reynolds PN Chia C . Preclinical interstitial lung disease in relatives of familial pulmonary fibrosis patients. Pulmonology. (2023) 29:257–60. doi: 10.1016/j.pulmoe.2022.08.005 36216738

[71] Aburto M Aguirre U Arrizubieta MI Pérez-Izquierdo J Bronte O Gorordo I . Checking siblings of patients with idiopathic pulmonary fibrosis as a scheme for early disease detection. Ann Am Thorac Soc. (2021) 18:172–4. doi: 10.1513/AnnalsATS.202002-111RL 32881588

[72] Podolanczuk AJ Kim JS Cooper CB Lasky JA Murray S Oldham JM . Design and rationale for the prospective treatment efficacy in IPF using genotype for NAC selection (PRECISIONS) clinical trial. BMC Pulm Med. (2022) 22:446. doi: 10.1186/s12890-022-02281-8 36514019 PMC9746571

[73] Zhang D Newton CA. Familial pulmonary fibrosis: genetic features and clinical implications. Chest. (2021) 160:1764–73. doi: 10.1016/j.chest.2021.06.041 34186035 PMC8628177

[74] Cerri S Manzini E Nori O Pacchetti L Rossi L Turchiano MG . Genetic risk factors in idiopathic and non-idiopathic interstitial lung disease: similarities and differences. Medicina (Kaunas). (2024) 60:1904. doi: 10.3390/medicina60121967 39768847 PMC11677115

